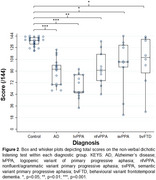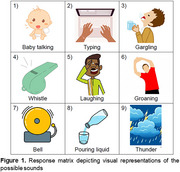# Non‐verbal dichotic listening: a new, cross‐cultural central hearing test for dementia

**DOI:** 10.1002/alz70857_100128

**Published:** 2025-12-24

**Authors:** Chris JD Hardy, Benjamin A Levett, Jessica Jiang, Lucy B Core, Sophie A Froud, Anna Volkmer, Nehzat Koohi, Doris‐Eva Bamiou, Charles R Marshall, Jason D Warren

**Affiliations:** ^1^ University College London, London, United Kingdom; ^2^ Queen Mary University of London, London, Greater London, United Kingdom

## Abstract

**Background:**

Difficulties with auditory scene analysis (the process of parsing the auditory environment into its constituent sound objects) are an early feature of Alzheimer's disease. Previous research has shown that tests of auditory scene analysis and other aspects of central (i.e. brain‐related) hearing can differentiate between people with Alzheimer's disease and healthy controls, but many of these tasks are based on speech signals, making extensions to and comparisons across other languages and cultures difficult. Here we introduce a new non‐verbal dichotic listening test (NVDLT) that addresses these limitations.

**Method:**

We administered the NVDLT to 17 patients with amnestic Alzheimer's disease, 13 with the logopenic variant of primary progressive aphasia (IvPPA), 10 with the nonfluent/agrammatic variant of primary progressive aphasia, 10 with the semantic variant of primary progressive aphasia, eight with the behavioural variant of frontotemporal dementia, and 27 age‐matched healthy controls. On each trial, participants were presented with two pairs of universally familiar non‐verbal sounds (baby babbling, typing, gargling, whistle blowing, laughing, groaning, bell ringing, pouring liquid and thunder), with one sound from each pair presented to the left ear as the other sound was presented simultaneously to the right ear. There were nine possible sounds and the participant was required to respond by pointing to the sounds they heard on a visual matrix (Figure 1). In analyses we adjusted for peripheral hearing, measured using pure‐tone audiometry, as well as age, sex, and Mini‐Mental State Examination score.

**Result:**

All patient groups performed significantly worse on the NVDLT than healthy controls (Figure 2). However, there were no significant differences between the dementia subtypes. In receiver operating characteristic analyses, NVDLT score discriminated patients from controls very well (AUC = 0.93), with outstanding performance in subgroup analyses between controls vs Alzheimer's disease (AUC = 0.99), and controls vs lvPPA (AUC = 1.0).

**Conclusion:**

Our findings highlight the importance of assessing central hearing in people with neurodegenerative diseases and suggest that the NVDLT is a promising and sensitive probe of dementia, particularly in those syndromes likely to be underpinned by an Alzheimer's pathological process and across culturally diverse older populations.